# Connecting to the Heart: Teaching Value-Based Professional Ethics

**DOI:** 10.1007/s11948-020-00216-2

**Published:** 2020-04-16

**Authors:** Roel Snieder, Qin Zhu

**Affiliations:** 1 Office of Academic Affairs, Colorado School of Mines, Hill Hall 206A, Golden, CO 80401 USA; 2 Division of Humanities, Arts, and Social Science, Colorado School of Mines, Stratton Hall 306, 1005 14th Street, Golden, CO 80401 USA

**Keywords:** Value-based ethics, Professional ethics education, Self-reflective pedagogies, Engineering education, Personal ethics

## Abstract

Engineering programs in the United States have been experimenting with diverse pedagogical approaches to educate future professional engineers. However, a crucial dimension of ethics education that focuses on the values, personal commitments, and meaning of engineers has been missing in many of these pedagogical approaches. We argue that a value-based approach to professional ethics education is critically needed in engineering education, because such an approach is indispensable for cultivating self-reflective and socially engaged engineers. This paper starts by briefly comparing two prevalent approaches to ethics education in science and engineering: *professional* (teaching professional ethical standards, including codes of ethics) and *philosophical* (teaching ethical theories and their applications in professional settings). While we acknowledge that both approaches help meet certain ethics education objectives, we also argue that neither of these is sufficient to personally engage students in authentic moral learning. We make the case that it is important to connect ethics education to the *heart*, which is extensively driven by values, and present a value-based approach to professional ethics education. We provide some classroom practices that cultivate a safe, diverse, and engaging learning environment. Finally, we discuss the implications of a value-based approach to professional ethics education for curriculum design and pedagogical practice, including opportunities and challenges for engineering faculty eager to incorporate value-based inquiry into their classrooms.

## Introduction

When our true values, virtues and competencies flow through us, whatever the choice, then transformative power—life power—is ours as well. We are enlivened, as is everything our choice touches. (Sinetar 1988, p. 159)

Ethics education for engineers is of critical importance because engineering increases our power over the world, yet knowledge of engineering does not come with a recipe on how that power is to be responsibly stewarded. In other words, despite that engineering has consequences for humans and the environment—which largely is the reason humans engage in these activities—engineering usually does not come *explicitly* with the values or “the moral compass” that guide the wise application of the engineering power. The history of engineering has generated ideologies, such as technocracy and meritocracy, that disengage engineers from reflecting on the sociopolitical nature of their identities and practices (Slaton 2015). This allows engineers to distance themselves from the political and value aspects of their work, which can potentially lead engineers to design artifacts that enable serious issues, such as social and environmental injustice (Karwat 2019). Approaches to engineering ethics education are often instrumentalist, especially when ethics is defined in terms of “ethical skills, competencies, and practical tools (e.g., codes of ethics, ethical theories, and other decision-making tools such as ‘line-drawing’)”, expected to be applied by engineers to solve professional ethical dilemmas (Zhu and Jesiek 2017). Engineers are, then, assumed to be instrumentalists who are mainly interested in the effectiveness and proficiency of ethical problem-solving. The self-reflective dimension of engineering—where engineers consciously reflect on the values embedded in their work and its broader impacts—is often absent from the practice and education of engineers.

Mitcham (2014) makes the case that society needs a critical reflection on the purpose for which we use engineering. He invites engineering educators and future engineers to carefully examine the following “big” questions: What is the world that we seek to create? How does engineering contribute to this? Who benefits from these choices, and who is hurt? Mitcham (2014) considers these questions to be the true grand challenges for engineering, but current engineering curricula often provide limited (if any) space for students to engage in them. Karwat (2019) provides a more extensive list of similar, thought-provoking questions.

Capra notes that “it is generally not recognized that values are not peripheral to science and technology but constitute their very basis and driving force” (Capra 1996, p. 11). In other words, it is unavoidable that values—defined as that which we hold to be important—are a driver of individual and collective behavior (Haidt 2013; Roeser 2012). The Conscious Capitalism movement (Mackey and Sisodia 2014) shows that corporations in a capitalist society *can* transcend the value of generating monetary profit, while being profitable. But how can we expect that students, as future employees of corporations and universities, exercise their profession in ethical ways when they have not learned to discover and articulate what their values are, what it is they want to stand for?

If values are such an important driver of individual and collective behavior, should we not let engineering students reflect on the values of engineering, and on the roles that these values will assume in their careers? Dominant approaches to engineering ethics education provide limited opportunities for students to reflect on their own values, meaning, and commitments that will impact their long-term professional development. The traditional approaches to teaching engineering ethics are not sufficient for allowing students to problematize and engage in the true grand challenges for engineering in the way in which they practice their profession.

There are examples that demonstrate the limitations of traditional approaches to ethics education and justify the urgency of an approach that focuses on values, meaning, and a moral compass. Wike (2001) argued for using values as the basis for engineering ethics education, but she did not connect such a value-based approach to prevalent engineering ethics education approaches. Barnwell (2016) argues that students have largely lost their moral compasses, and states that “the pressures of national academic standards have pushed character education out of the classroom.” Even though he refers to K-12 education, one can still argue that the same holds for engineering universities, which focus extensively on the development of technical and disciplinary skills while overlooking the character development and personal growth of future professionals. The absence of a moral compass is further demonstrated in the “2012 Report Card on the Ethics of American Youth” (Josephson Institute for Ethics 2012), which states that although 98% of 9–12 graders say that “it’s important for me to be a person with good character”, 40% of the same group also say that “a person has to lie or cheat sometimes in order to succeed.” Accompanying this loss of a moral compass is the worrisome state of the mental health of college students. According to the American College Health Association (2018), 41% of college students felt at some moment in the last 12 months so overwhelmed by depression that it was difficult to function, while 12% seriously considered suicide. There are many factors contributing to the poor state of mental health of students, but a loss of meaning and purpose is a recognizable one (Martin 2000).

We argue that engineering students need more for their development and wellbeing than solely scientific and technical education. College students are in the process of developing their professional identities, and values often help give shape (in explicit and implicit ways) to these identities. Studies in the history and philosophy of science have shown that values deeply influence the choices of scientific methods, concepts, assumptions, and questions and the ethical decision-making of practicing engineers (Elliott 2017). Roeser (2012) argues that as design is not value-free and values and emotions are integral for engineering decision-making, they should be part of the development of professional identities of engineering students. Similarly, Troesch (2014) advocates for a phenomenological approach to ethics that invites students to consciously explore and reflect on the everyday, lifeworld experience of being an ethical engineer. In other professions such as business, Arce and Gentile (2015) point out that values should be part of a business education and they introduce the concept of *Giving Voice to Values*, while Brophy (2015) argues for incorporating spiritual values in business. Therefore, responsible educators and policymakers need to carefully consider at least two questions: (1) what is the role that higher education plays in the development of professional identities? and (2) what role can or should ethics education play in this development?

To a large extent, ethics education in engineering in the United States often adopts a *professional* approach that places a strong emphasis on engineers’ *individualistic* obligations that prohibit misconduct behaviors and prevent their practices from harming the public (Conlin and Zandvoort 2011). In this view, engineers are portrayed as *rational* and *autonomous* humans who “act individually and independently in relation to client[s]” as it is only the professionals who are “able to act based on adequately developed knowledge” (Luegenbiehl 2004, p. 59). Personal traits such as emotion, virtues, and commitments are sometimes invisible in engineering education or are considered irrelevant (Davis 2015; Harris 2008). In summary, the traditional professional approach to engineering ethics education often assumes that engineers are isolated, rational, and autonomous human beings and engineering as a profession needs to be *depersonalized*.

Engineering ethics textbooks and other teaching materials often start by making a clear distinction between *personal* and *professional ethics*.^1^ Here the assumption is quite straightforward: to be professional is to be impersonal or disinterested. Such a depersonalizing tendency in professional education can be traced to early works in the sociology of science. For example, according to Robert K. Merton, scientists are supposed to “engage in an impersonal and disinterested search for the truth” and “academic organizations should embody a form of organized skepticism that rectifies individual shortcomings” (Hansson 2017, p. 10). Merton’s work has exerted profound influence on the professional education of scientists (and engineers) in the United States. More recently, scientists Kipnis and South argue that “personal values are not relevant to issues in professional ethics” (Kipnis and South 2000, p. 13), as they are too subjective (e.g., akin to personal color preferences) to be considered as part of professional ethics applicable to all engineers no matter where they are from. However, a separation between personal values and professional behavior amounts to a lack of integrity, a concept which, according to Snieder and Schneider (2016), implies that all the elements of our life support each other and that we always are a true person who upholds and out-pictures our values consistently. This is what Palmer (2004) calls “living a life without compartments.” Living in integrity thus implies that our personal and professional values are aligned.

The main point of this paper is that ethics education can be an essential component of the broader development of students, and that ethics education needs to go beyond a depersonalized approach that focuses on teaching only the professional (e.g., what should be done by following codes of ethics), intellectual or philosophical (e.g., ethics as a purely intellectual or philosophical exercise) aspects of morality. Engineering students should be perceived as “flesh-and-blood” persons (Ames 2011) with their own values, goals, and commitments. We, as their teachers, need to make ethics education alive and connected to issues that students hold as important. To achieve this goal, ethics education needs to help students develop capacity for self-reflection in the process of “personal analysis of one’s beliefs, actions, and outcomes of those actions” (Karwat 2019, Self-Refection for Activist Engineering section, para. 2). Engineering educators and university leaders, thus, need to view ethics education as an important contributor to the growth of future professionals instead of another mandatory component of the engineering academic curriculum.

In the following, we first briefly compare the two prevalent approaches to ethics education in science and engineering: *professional* (teaching professional ethical standards including codes of ethics) and *philosophical* (teaching ethical theories and their applications in professional settings). We, then, make the case that it is important to connect ethics education to the *heart*. We use the word *heart* to describe the feelings, convictions, and passions that humans have and value. Arguably, these are also unique and intuitive ways of expressing values that complement our rational ways of thinking. For example, a person may feel ashamed if she fails to live up to or value something (Mulligan 2010). Such shameful feeling indicates that she values something important for her life. Subsequently, we provide ideas on how to incorporate values into ethics education for engineers in order to make ethics education personal, meaningful, and relevant. Finally, we conclude with a discussion of the challenges and opportunities educators may encounter while teaching value-based ethics.

## Dominant Approaches to Professional Ethics Education: Professional and Philosophical

In practice, the goals of ethics education vary among institutions, the scopes of classes, and even instructors. One prevalent method is the *professional* approach, which is often concerned with teaching professional ethical standards and how to contextualize and comply with these standards in professional practices. This is the case, for example, when students review and sign an ethics code, a popular extracurricular activity at engineering universities, an activity that often is superficial. Another example is the way in which the research ethics training requirement of the National Science Foundation is implemented at many institutions (National Science Foundation n.d.): students take an online training, pass the online test, and then they are assumed to have received adequate ethics education, regardless of the degree to which they have internalized the material, or have a personal commitment to apply what they have learned in their future careers.

**Fig. 1:**
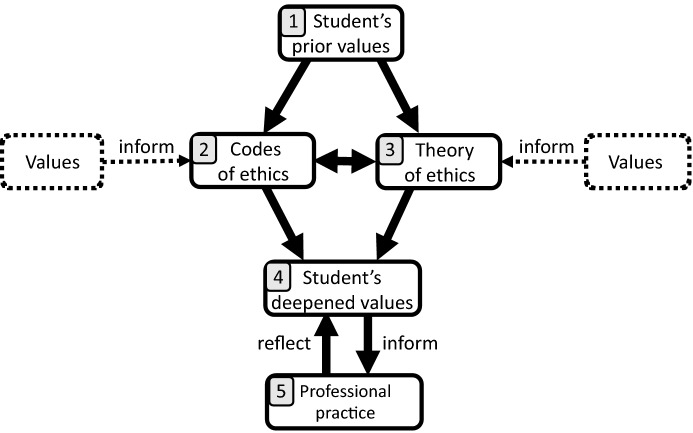
Diagram of different levels of ethics education and the connection of values

We use Fig. 1 to illustrate the different approaches to ethics education and how they are related to values. Before students receive any engineering ethics education, they have a set of preexisting values. This is indicated by box 1 in Fig. 1. These prior values may be deep or shallow, and they may or may not be connected to engineering practice. Similarly, codes of ethics (box 2 in Fig. 1) have been informed by values that the engineering community holds. Yet in presenting these codes of ethics to students, these underlying values may not be explicitly articulated.

The second dominant approach to professional ethics education is the *philosophical* approach, which teaches students the historical, social, or philosophical viewpoints of ethics. We label this the theory of ethics in box 3 of Fig. 1. In this approach, students gain a broader and deeper understanding of the ethical issues and of the ways to think about these issues. As indicated in Fig. 1, ethical theories are also informed by values. For example, social values (e.g., making a better world) may drive consequentialism; historical and/or religious values (e.g., moral duties derived from historical and religious traditions) may drive duty ethics; virtuous values drive virtue ethics; and the values of compassion drive the ethics of care. Yet these underlying values are often not made explicit and the conversation centers on philosophical argumentations.

Both approaches—professional and philosophical—serve a purpose of professional ethics education and may enrich students’ learning experience. However, they also have limitations that may prevent students from committing to a *lifelong* project of moral learning and development. In professional ethics education, reviewing the professional codes of ethics serves a purpose. Martin and Schinzinger (2005) argue that engineering codes of ethics may play at least eight different roles, and are expected to:

(1) serve and protect the public (they function as a commitment of engineering professionals to hold paramount the health, safety, and welfare of the public); (2) provide guidance (they provide some guidance on the professional responsibilities of engineers); (3) offer inspiration (they provide positive motivation for ethical conduct); (4) establish shared standards (they communicate the moral standards shared by all engineers to the public); (5) support responsible professionals (they provide engineers with some group backing and even legal support); (6) contribute to education (they can be used to facilitate discussions and reflections on professional ethical issues); (7) deter wrongdoing (they can be used as the formal basis to investigate ethical misconduct); and (8) strengthen a profession’s image (they can present a positive image to the public that engineering professions are ethically committed). (pp. 44–46)

Michael Davis, one of the most well-known advocators for codes of ethics, argues that codes of ethics are critical for the sustainable development of engineering professions. According to Davis (1991), there are at least four reasons why professionals should support their profession’s code:

First…supporting it will help protect them and those they care about from being injured by what other engineers do. Second, supporting the code will also help assure each engineer a working environment in which it will be easier than it would otherwise be to resist pressure to do much that the engineers would rather not do. Third, engineers should support their profession’s code because supporting it helps make their profession a practice of which they need not feel…embarrassment, shame, or guilt. And fourth, one has an obligation of fairness to do his part…in generating these benefits for all engineers. (p. 166)

Nevertheless, there are some caveats to be made with regards to the functionality of engineering codes of ethics. First, in order for codes of ethics to be efficacious they often require: (1) collective commitment (e.g., a code of ethics is a convention that a group of engineers agree on); and (2) personal commitment (e.g., joining a professional engineering society means that one promises to comply with its code of ethics and implicitly commit in certain ways required by the code of ethics) (Peterson 2020). This raises the question to what extent a code of ethics would still work in any of the following circumstances: (1) engineers only pay “lip service” when joining engineering societies and reviewing the code of ethics; (2) they do not commit themselves to the values underlying different articles of a code of ethics; (3) they treat the code of ethics mainly as a legal document rather than a source of inspiration; (4) they think anything is ethically acceptable insofar as it is not prohibited in the code of ethics. In order to make codes of ethics *really work*, we need more than just the written articles included in them. We need engineers to commit themselves to the *values*, as indicated in the left box of Fig. 1, that support the formulation and implementation of these codes of ethics.

Second, students may raise questions that challenge ethics codes. Here, we highlight some questions that have arisen from our own experience with teaching the code of ethics of the National Society of Professional Engineers (NSPE). Item 1 of the first “fundamental canons” reads “Hold paramount the safety, health, and welfare of the public” (National Society of Professional Engineers 2019). This raises questions such as: Should a professional engineer work for a company that develops weapons of mass destruction, or that produces foods or drinks without nutritional value, or which may be damaging for public health? Is it ethical to work for an employer that spreads misinformation about climate change? Much later in the NSPE Code of Ethics, in section III, item 2d it is stated that “Engineers are encouraged to adhere to the principles of sustainable development in order to protect the environment for future generations.” From some engineering specialties’ perspective, there is inherent tension between holding the safety, health, and welfare of the public *paramount*, and *encouraging* the protection of the environment. For instance, students in petroleum engineering may defend their profession by arguing that their everyday practice is *itself* ethical as it contributes to the ideal of serving the welfare of the public by providing a source of energy. At the same time, some feel puzzled by their difficulty in balancing the extraction and use of hydrocarbons with protecting the environment. We, as instructors, have been challenged when teaching the code of ethics to students in an authentic, reflective, and meaningful way, as we are sympathetic to the challenging questions raised and their struggle to internalize and harmonize the code of ethics with their *own* personal values.

Third, ethics codes may not always be sufficient to resolve dilemmas that arise in practical situations. As noticed by Lyes (1952),

ethics, in the sense of a code of ethics, is not a complete guide in decision-making, since our more difficult choices are among alternatives that arise when we cannot act strictly according to our professional code or when we are not sure how to apply the code to the immediate situation. (p. 12)

It is widely acknowledged that codes of ethics often have limitations that may affect their practical effectiveness in guiding ethical decision-making in engineering: (1) most codes are written in general wording and contain substantial areas of vagueness; and (2) codes of ethics can be misused or abused if not taken seriously, e.g., treated as “sacred documents” rather than documents open to public criticism (Martin and Schinzinger 2005).

Given these reservations and limitations of the ethical codes, it is not surprising that despite their crucial role in the history of the engineering profession, research has indicated that codes of ethics only play a limited role in ethical decision-making among practicing engineers. For instance, a survey conducted in 1983 by the journal *Chemical Engineering* showed that written codes of ethics had little impact on the professional ethical decision-making of chemical engineers (Luegenbiehl and Puka 1983). According to the survey, “although the American Institute of Chemical Engineers… has a code of ethics, this was almost universally ignored in determining the solutions to our survey problems [ethical cases and scenarios]. Fewer than a half-dozen [out of 4318] respondents even mentioned a code of ethics at all.” (Luegenbiehl and Puka 1983, p. 41).

The second approach to ethics education is based on the philosophy of ethics and the historical development of ethical thinking. The history of philosophical ethics is rich, and the distinction between utilitarian, deontological, and virtue ethics helps students understand the different drivers for ethical decision making. Yet, this approach to ethics teaching may reduce ethics to an intellectual exercise that focuses on the nuanced differences between different ethical theories.

Engineering ethics textbooks often start by introducing classical ethical theories such as deontology, consequentialism, virtue ethics, etc.^2^ However, consequentialism and especially deontology are more frequently used to analyze and discuss ethical cases in the classroom compared to virtue ethics (Harris 2008). Such an applied ethics approach does not often provide a clear justification for prioritizing certain mainstream ethical theories as the fundamental tools for ethical reasoning, and it tends to overlook other less prominent Western (e.g., ethics of care, feminist ethics, pragmatism) and non-Western (e.g., Confucian ethics, African ethics) ethical theories (Zhu and Jesiek 2017). Engineering faculty and students often find it difficult to apply general ethical theories and principles in specific cases since “there continues to be serious disagreement among philosophers as to which principles or set of principles should be used” (Luegenbiehl 2010, p. 149). Finally, some philosophers have found that applying different ethical frameworks may lead to different outcomes (van de Poel and Royakkers 2011). Nonetheless, the ability to reflect on and compare the outcomes generated by different ethical frameworks should be taught to students since it helps them understand the variety of ethical viewpoints and it further gives them insights into their own ethical drivers. This insight can be deepened when ethical theories are connected to the values that underlie these theories (the right box in Fig. 1).

Moral psychologists, such as James Rest, argue that there are multiple elements that contribute to ethical action: moral sensitivity (or recognition), moral judgment (or reasoning), moral motivation, and moral character (Rest et al. 1999). Arguably, teaching ethical theories might be able to improve students’ moral judgment (and maybe also their moral sensitivity). However, moral motivation and moral character are too complicated to be shaped exclusively by the knowledge of ethical theories. An engineer may be aware of ethical theories and the right course of action but lack motivation and/or character to *actually do good and take the right action*.

In other words, knowing what is right and wrong does not necessarily lead to ethical action. People need to be personally committed to the moral judgment they have made. Engineering students may be only interested in the intellectual part of these theories but not apply them to their own situations. In other words, they are not engaged. If they *truly* believe the consequentialist theory, do they still want to invent unhealthy drinks or food that can potentially bring long-term welfare concerns to a large part of the population? Nevertheless, we are not arguing that ethical theories should not be taught. What we caution against is that ethical theories are taught in a purely skill-based way: students use the ethical theories to solve a hypothetical dilemma and then set the theories aside and return to their technical decision. In such an approach, ethical theories are considered as first aid tools, useful and visible only when engineers and their companies face financial and legal threats. A worse case is that engineers and engineering students misuse ethical theories (sometimes oversimplified versions they learn in a relatively limited amount of time in the classroom or online) to justify wrongdoings.

Teaching ethics based on the codes of ethics helps students be aware of general ethical issues they may encounter in their professional life, while teaching the philosophy of ethics and ethical theory helps expand their ethical perspective. Both approaches are worthwhile, but as argued above, they both have their limitations (as shown in Fig. 1, codes of ethics and ethical theories are informed by values that may never be mentioned in the classroom). In the next section we explore what is missing in these approaches to ethics education.

## Connecting to the Heart

We live in a society preoccupied with achieving. Ethics teaching based on the codes of ethics tells us the rules we must follow while we are doing the achieving, but it does not provide opportunities to reflect on *what it is we seek to achieve*. Teaching philosophy of ethics provides students with different models for ethical thinking and a broader and deeper perspective, that may be disconnected from the values and passions that many students have. Limiting professional ethics education to codes or philosophy of ethics is a symptom of the achievement-oriented approach that many universities and their faculty pursue. As Brooks (2019) states,

Students are engaged in critical thinking to doubt, distance, and take things apart, but they are given almost no instruction on how to attach to things, how to admire, to swear loyalty to, to copy and serve. The universities, like the rest of society, are information rich and meaning poor. (p. 194)

This is, in particular, the case for engineering schools with their focus on technical and intellectual topics. While going through such an education, students may hide their sense of purpose and meaning, or let it submerge to live the dream of achievement. The cultures of engineering education tend to instill a kind of “sociotechnical dualism” that invites students to separate the technical from the social (Slaton 2015). An underlying message of such ideology is that students who raise social issues, such as those involving values and meanings around engineering, will be considered as unprepared for service to the engineering profession. Erin Cech’s (2014) research has shown that students’ support for public welfare decline over the course of their engineering education. One explanation is that engineering programs embed the often unspoken premise into their curriculum that non-technical cultural beliefs (e.g., ethics and responsibility, social welfare) *are important but less important* than math and science skills therefore prioritize the technical over the non-technical, including concerns about social and environmental implications, ethics, values, and meaning.

Adolescents naturally ask questions such as, What is my purpose? What am I here to do? How can I make the world a better place? One can argue that such questions are also ethical questions, as they relate to how thoroughly our personal and professional lives make a positive difference in the world. Universities, whose educational mission is to help adolescents grow into responsible and mature professionals, should stimulate students to ask such questions and find meaningful answers. Many academic programs leave no space for such broad and big questions (see the quote of Brooks above) and since the answers don’t come easily, many of us may give up. Ethics education is the perfect vehicle to do this work with students, as we are reminded, by the poem *At the End of the Day, a Mirror of Questions* by O’Donohue (2008), from which we quote the following lines:

What differences did I notice in those closest to me?

Whom did I neglect?

Where did I neglect myself?

What did I begin today that might endure?

How were my conversations?

What did I do today for the poor and the excluded?

….

What did I avoid today?

From the evidence—why was I given this day? (p. 98)

Teaching an undergraduate class on “Science and Spirituality” annually to about 25 undergraduate engineering students has shown us that students love thinking about questions like these. Integrating such questions into an ethics curriculum teaches students that it is natural and healthy to bring these questions to the life of the professional.

Note the question “what did I avoid today?” in the poem. We tend to focus in ethics education on staying away from the don’ts, on following rules, and we tend to not pay much attention to what *would* be the right thing to do. In fact, we think that the main ethical question to ask ourselves is *What do I give my energy and my talents to?* This is an ethical question, because the choice of what we give ourselves to determines how we contribute to humanity and/or the environment. This question focuses both on what the right action is, and on what a good person is supposed to do. It has a bearing on the meaning and the fulfillment that we seek. It determines what difference we will make, and who or what benefits from or is hurt by our actions. These are deep and meaningful ethical issues that young people love to engage in. These issues are related to our values, and it is for this reason that we make the case to supplement and enrich traditional engineering ethics education with value-based ethics.

## Toward Value-Based Professional Ethics Education

What do we value? That which we deem to be important. We are more likely to make choices that support that which we value. Our values are shaped by factors such as our history, upbringing, past experiences, faith, and education. Values come both from within and from outside whereas rules are externally imposed (e.g., codes of ethics were developed by professional societies). In general, we are more likely to break rules when they are not aligned with our values.

Ideally, rules are based on values. If we embrace the value that supports a rule, then we are like to follow the rule, but adherence to rules, whose value we question or simply don’t understand, leads to actions governed by fear of punishment. In a college environment, this may apply to the rule to not plagiarize. We often fail to tell students what values this rule seeks to support, and it should not come as a surprise that students’ choice of whether to plagiarize or not is based on the chance of being caught (Holsapple et al. 2012).

Real-life situations are more complicated, as values may often conflict. For example, when designing a car, how does one balance the value of passive safety for the occupants with the value of a clean environment supported by a lighter, less polluting automobile? If we only look at engineering codes of ethics, we may be confused by what we mean by *the public* and its health, safety, and welfare. Does that refer to the occupants of the car, or to others who will be affected by a more polluted environment? Dilemmas such as these don’t have easy answers, but without knowing how to value the different alternatives, one has no map to navigate by for making wise decisions. Incorporating values into decision-making thus does not solve the problem of dealing with conflicting considerations—as we noted earlier with codes of ethics or with ethical theories—but incorporating values into such conflicting considerations assists our choices to be driven by what we consider to be most important.

It may appear that values are of limited use for ethical decision-making as critics may argue that there is a personal and subjective component to them as they are often shaped by our past, experience, upbringing, and for many, by our faith. In that respect, somebody might hold values that others find repugnant, which is a challenge in the classroom when a student supports such values. For example, many of us now frown on the value of racial supremacy, but at the time of Apartheid this was a fairly accepted value in some circles. A deeper analysis of our values and of their consequences would include the following:

- A conversation to make us accountable for our values. This involves an analysis to find out what core beliefs or core values are most important. One may discover in the process that what we think of as a value is really an expression of fear or personal interest, as might have been the case for some of the proponents of Apartheid.
- Apply some intuitive moral tests on the consequences of these values, using for instance, Davis’s Eight Moral Tests (2014). These tests pose simple and intuitive questions to guide students through their reflections on their decision-making. For instance, we can use the “harm test” to examine whether the implementation of a specific value, such as the value of racial supremacy, would actually harm people. Or, we can use the “rights test” to examine whether the value of racial supremacy would violate anyone’s right, especially a human right.

Whitbeck (2011) points out that it is crucial for engineers and engineering students to reflect on their own values in engineering practice by asking themselves the following questions: (1) in deciding to enter engineering, what value judgment did you make (or others, such as parents and guidance counselors, make for you)? Have those values changed as you have learned more about engineering? (2) what makes a good engineer and good engineering? and (3) what reasons can you give to support your value judgments about engineers and engineering? Asking these self-reflective questions about values and their role in professional development is a critical lifelong learning skill for professional engineers.

We now have over seven years of experience with value-based ethics teaching to engineering students (group size 20–40) in two undergraduate classes *Research, Values, and Communication* and *Science and Spirituality* and two graduate classes *Introduction to Research Ethics* and *The Art of Science*. We have not encountered a single case where students proposed values that were considered to be outlandish or repugnant by us or by their fellow students.

Some argue that ethics teaching must be value-neutral. In other words, ethics instructors should not try to impose ethical theories on students (Rachels and Rachels 2019). This is, perhaps paradoxically, what we advocate. When we speak about value-based ethics, we don’t mean that we impose values on students. That would actually amount to a preaching approach, which is exactly what we want to avoid. Instead, we consider it our task to help students discover and reflect on what their values are. The codes of ethics and the ethical theories may support this process. But we advocate asking students to take the next step and to go on a discovery tour to find out what their *own* values are. In this process, students often find after reflection that their values differ from what they originally thought. For example, many students state at the start of one of our courses that they have a utilitarian point of view: the greatest good for the greatest number of people. During class conversations students discover that they have vague ideas of who should or not be included in *the greatest number of people*. They further discovered that they were, for example, much more driven by virtues or by their faith than they thought they were, and they rediscovered the values they truly upheld. Callahan (1980) articulates this process with the following words,

Self-knowledge is fundamental because feelings, motives, inclinations, and interests both enlighten and obscure moral understanding. In the end, individual selves, alone with their thoughts and private lives, must wrestle with moral problems. This sort of struggle often forces one to confront the kind of person one is, to face one’s character and integrity and one’s ability to transcend narrow self-interest to make good moral decisions. (p. 15)

It is our job as ethics educators to assist students on this journey of self-knowledge, and exploration and articulation of values. With the addition of value-based ethics to engineering ethics education, students get the opportunity to hone and deepen their values, as indicated in box 4 of Fig. 1. These deepened values are influenced by the prior values of the student, the values that inform the codes of ethics and ethical theories, the reading, conversations, and exercises in class, and the synthesis that the student makes of these considerations and experiences. The ultimate goal is for students to bring these deepened values to their professional engineering practice (box 5 in Fig. 1), and an intentional interplay between their values and engineering practice can help engineers further reflect on and deepen their values.

## Implications for Curriculum Design and Pedagogical Practice

Teaching value-based ethics is a challenge for ethics educators because the topic of values is personal, and sometimes it can bring up strong emotions. It is much easier to discuss codes of ethics and ethical theories because this can be done with an intellectual detachment that keeps personal values and priorities at bay. But such an approach hides the reality that many of us have strong feelings about our values, and that we need to share these feelings and confront the issues that arise in conversations. In this process, the class becomes personal. We argue that it needs to be personal if we want to have any hope that ethics education will lead to wiser and more responsible personal and professional choices.

In order to channel this process in a positive way, it is essential to spend time early in the course on creating a class atmosphere where students feel safe. Students can only feel safe in an environment that is respectful, and thus in our classes we spend significant time on the concept of respect. The word *respect* is both a noun and a verb: one can have respect, and one can respect a person. Respecting differences in worldviews, opinions, priorities, and values is essential, but we also explain that respecting such differences does not mean that one condones them or needs to agree with other points of view. Part of having respect is that we let go of the idea of wanting to be right or that others agree with our point of view (both are often valued in philosophical debates). After all, if we value the identities of ourselves and of others and assume good intentions, it is much easier to respect differences.

Nevertheless, readers may wonder whether we are self-contradictory as we discussed our support for value-neutral ethics pedagogy while imposing the value of respect to create a safe learning environment. Values, such as respect, are indispensable for building norms that allow students to participate in meaningful learning and (inter)acting in the classroom as a community. We call these values for building communal norms *cardinal values* and, when introducing them in our classroom, we do not force students to comply. Rather, we take a personal approach to learning and invite students to reflect on: (1) why these values are critical for their own participation in a community like the classroom; and (2) how these values can potentially support their journey toward the discovery of and reflection on their own values.

Part of the creation of a safe class environment is a conversation about debate versus dialogue. The book *On Dialogue* by Bohm (1996) offers great reading material for this topic. Debate is usually not about convincing but verbal out-maneuvering the other party. Through debate contests in high-schools or colleges, and through the current political culture, students are familiar with the concept of debate, and their default behavior in an ethics class is to debate ethical issues. But since debate is not really aimed at changing our views, it does not contribute much to the exploration of values. In addition, the confrontational or aggressive nature of debates makes many students, especially introvert ones, shut down. But in an atmosphere of dialogue there is an exchange of ideas, and the possibility exists that a third viewpoint arises. Therefore, a class atmosphere of respect and dialogue is essential, especially when a difference in views is likely to arise.

According to Bronowski (1956), science is built on two pillars or values: honesty and dissent. Honesty is important because it is often impossible to fully replicate the work that others have done, therefore we must be able to trust our fellow scientists and engineers that they act in good faith. Dissent is essential to the advancement of science and engineering because the questioning of common knowledge spurs further innovation. Honesty and dissent are also key ingredients of a well-functioning democracy, and students appreciate this analogy. Additionally, we stress the importance of disagreeing respectfully, and provide guidelines for respectful disagreement in our graduate and undergraduate classes.

We also have a class conversation in which we point out how little students know each other. Students sometimes bring up fictitious scenarios to illustrate a point. These scenarios may, unknowingly, generate strong emotions because they may relate to a traumatic or hurtful experience of another student, or bring up strong feelings of guilt or shame. Therefore, we ask students to be cognizant of how little they know about the personal life of their colleagues and to be sensitive to not bring up examples that may be confrontational. Being sensitive to the pain of others is another example of demonstrating the value of respect.

Another reason why teaching value-based ethics is challenging is that when we ask students to take the conversation to a personal level and to share their views in class, we—as teachers—have to be engaged and share our own stories and values from a personal point of view as well. That may include sharing that we don’t have all the answers, that some dilemmas are truly difficult to resolve and that we cannot provide resolutions. Perhaps we can share our own doubts, possibly our mistakes. In other words, we must be willing to be vulnerable, which is difficult for many engineering faculty and professional engineers. In fact, we often receive students’ appreciation of our willingness to be authentic and vulnerable in teaching evaluations. Not every engineering teacher has this mindset (Troesch 2014), posing restrictions as to who could teach value-based ethics.

We do a number of self-reflective exercises in class to help students discover and reflect on their values. Here we introduce three examples and additional examples can be found in Troesch (2014). The first is the *moral exemplars exercise*, that one of us learned at a local church. When we ask students what their values are, they often cannot give a clear answer, but most have role models whom they admire. So we ask students to write down the names of seven people (i.e., exemplars) they consider as positive role models. These can be scientists or engineers (e.g., Albert Einstein); famous public figures (e.g., Martin Luther King); friends (e.g., postdoctoral researcher Tom in our lab); family members (e.g., my grandfather); or they can be fictional characters (e.g., Frodo Baggins). Next, students list three character traits for each of their exemplars. This provides them with a list of up to 21 positive character traits. Each student picks those that are most meaningful, and often these are the traits that are most frequently selected. Since it is easier to think of moral exemplars than of abstract concepts, this exercise helps students get clarity on their values.

The second example is the final class project *personal ethics statement*. These are presented in the last class as the ethical values by which each student wants to lead their personal and professional life. We give students much freedom in how they present their statement; they can write a piece of text, a poem, create and sing a song, make a collage, or find another way of expression. We have a class conversation about each statement to gain a deeper understanding of the values presented. Students take this project very seriously and are willing to make the articulation of their ethical values personal and heartfelt. Often the presentations are quite emotional. We admire our students for making their personal ethics statement so heartfelt and passionate.

The third example is the *ethics autobiography* employed by one of us in his honors class on *Science, Technology, and Confucian Ethics*. An ethics autobiography is one’s personal account of her own moral experience. It has been widely used in health and psychological science programs to train future professionals to develop moral sensitivity and awareness of how their own personal values shape the processes by which they adopt professional practices and cultures, i.e., professional acculturation. Bashe et al. (2007) discuss how to use the ethics autobiography to train clinical psychologists. They assign a reflective writing assignment at the beginning of the semester and ask students to respond in writing to self-reflective guiding questions. At the end of the semester, students revise their ethics autobiography by incorporating their reflective learning experience throughout the semester. Bashe et al. (2007), some of the guiding questions students use as writing prompts include:

- What is your idea of right and wrong personal behavior, and where does this conception come from?
- What did you learn from your family of origin about right and wrong?
- What do you recall were the messages about ethic or cultural groups different from yourself, and how they see right and wrong?
- What is your idea of right and wrong professional behavior, and where does this conception come from?
- What formative experiences account for how you live your life?
- What experiences have you had in the field, and what ethical dilemmas have you already encountered?
- What professional ethics in the field are most compatible with your own personal values, and which professional ethics are least compatible?
- What aspects of this profession strike you as being “not intuitive”?
- What are your top three values, and where do they come from?
- What are three personal needs that you think match well with the profession?
- What are three personal needs that you think might conflict with the profession?
- What morals are most important to you, and where do they come from? How do these align with or conflict with the ethics code and professional standards?
- How might the alignment or conflict influence your work with clients or students? (p. 62)

An ethics autobiography has certain notable strengths that can complement other traditional ethics pedagogies. It can improve students’ self-reflection and moral sensitivity to the ethical significance of professional practice. It can also increase students’ awareness of their own values, assumptions, and even biases and the ways in which these affect their everyday decision-making. Because it can make students emotionally engaged and invested in moral learning, ethics becomes relevant and personal. Finally, it integrates multiple required student learning outcomes of the ABET (incorporated as the Accreditation Board for Engineering and Technology, Inc.) (2018) (e.g., ethics, communication, contextual and critical thinking, lifelong learning) in one writing-intensive exercise.

## Discussion and Conclusion

The main point we want to make is that since our decisions are affected by our values, it is important to enrich ethics education with components that assist students to hone and deepen their values. As values have communal and personal aspects, we need to bring a personal element into ethics education (Roeser 2012).

Traditional approaches to teaching ethics have their own merits. Codes of ethics (box 2 in Fig. 1) communicate professional expectations, and they show what ethical issues students may encounter in their professional lives. Teaching ethical theories and the history and philosophy of ethics (box 3 in Fig. 1) shows students that there is more than one way to think about ethics. This helps students understand a) why others may think differently, and b) discover how different ethical approaches can help their own ethical reasoning. But if ethics education does not go beyond these two approaches, there is a risk that ethics remains an intellectual abstraction disconnected from the students’ personal values. By incorporating values into ethics education, the topic of ethics becomes personal at a visceral level (box 4 in Fig. 1), helping students apply what they learned to reflect on the ethical decisions they encounter in their professional and personal lives (box 5 in Fig. 1).

We acknowledge that the codes of ethics and ethical theories are informed by values (the left and right boxes in Fig. 1), but the values that students ultimately develop (box 4) may differ from the prior values that students had (box 1), or from the values that inform codes of ethics or ethical theories. When done well, value-based ethics teaching assists students in the continued, life-long development of their values and their awareness of and their commitment to these values. We consider this to be an essential element of educating well-rounded responsible engineers.

The proposed work on value-based engineering ethics education has a bearing both on the engineering practice and on the conduct of research. In other words, we believe the values underlying, supporting, and justifying rules or codes are more fundamental and crucial than the rules or codes themselves. Thus, a value-based approach to ethics education can help us reframe concerns in engineering ethics education in the following ways:

- If we value *honesty*, then we don’t falsify data.
- If we value *integrity*, then we present our research truthfully without bias.
- If we value *sharing appropriately*, then we invite the right colleagues to be co-authors.
- If we value *giving credit where credit is due*, then we don’t plagiarize.
- If we value *fostering the growth of others*, then we are nurturing and compassionate mentors.
- If we value *loving others as we love ourselves*, then empathy and compassion come easily.
- If we value *making the earth a better place*, then we choose a career that serves the safety, health, and welfare of the public and the health of the environment.

Teaching value-based ethics is challenging, because the topic becomes personal, students are more involved, and strong feelings and emotions may come into the classroom. It is, thus, essential to create a class atmosphere in which students feel safe. If we ask students to apply the class to their own life and to share their values, we ask them to be vulnerable. If we ask this of students, it is important that as teachers we are also willing to be vulnerable, for example, by sharing our doubts or past mistakes, or by presenting our personal ethics statement. Value-based ethics teaching is more taxing than teaching ethics from an exclusively intellectual and academic point of view. But the reward of doing so is that students love thinking about their life’s purpose, and to connect their professional ambitions with meaning, personal growth, and values. And if we do that as ethics educators, we become guides that help students discover their values and priorities rather than academic professors who go through yet another dry intellectual topic.

## Footnotes

1 In the engineering ethics literature, there are other ways to distinguish different approaches to engineering ethics education. For instance, Herkert (2005) distinguishes micro ethics and macro ethics in teaching engineering ethics: (1) micro ethics: the responsibilities of individual engineers/professionals; and (2) macro ethics: the collective, social responsibilities of engineers and societal decisions about technology. Both micro and macro ethics in Herkert’s sense would belong to the category of professional ethics in our paper as they exist in the professional environment of engineers. Personal ethics in our paper is different from Herkert’s two approaches as it is mainly focused on the values, meanings, and commitments derived from engineers’ “non-professional” experiences (e.g., an engineer’s own value system may not be captured by her individual professional responsibilities which are often defined not by herself but by the profession and its cultures).

2 For instance, Vesilind and Gunn’s book *Engineering, Ethics, and the Environment* (1998) considers ‘‘applying’’ classical ethical theories as a way for engineers to practice environmental ethics. A few other examples that propose applying classical ethical theories include Mitcham and Duval’s book *Engineering Ethics* (1999) and Catalano’s book *Engineering Ethics: Peace, Justice, and the Earth* (Second Edition) (2014).
